# Effects of CPAP and FiO_2_ on respiratory effort and lung stress in early COVID-19 pneumonia: a randomized, crossover study

**DOI:** 10.1186/s13613-023-01202-0

**Published:** 2023-10-17

**Authors:** Lorenzo Giosa, Patrick Duncan Collins, Martina Sciolla, Francesca Cerrone, Salvatore Di Blasi, Matteo Maria Macrì, Luca Davicco, Andrea Laguzzi, Fabiana Gorgonzola, Roberto Penso, Irene Steinberg, Massimo Muraccini, Alberto Perboni, Vincenzo Russotto, Luigi Camporota, Giacomo Bellani, Pietro Caironi

**Affiliations:** 1 Department of Critical Care Medicine, Guy’s and St. Thomas’ National Health Service Foundation Trust, St. Thomas’ Hospital, Westminster Bridge Road, London, SE17EH UK; 2 Centre for Human and Applied Physiological Sciences, School of Basic and Medical Biosciences, King’s College London, London, UK; 3 Department of Pulmonary Medicine, AOU S. Luigi Gonzaga, Orbassano, Turin, Italy; 4 University of Turin, Turin, Italy; 5 Department of Anesthesia and Critical Care, AOU S. Luigi Gonzaga, Orbassano, Turin, Italy; 6 Department of Surgical Sciences, University of Turin, Turin, Italy; 7 Department of Anaesthesia, Intensive Care and Emergency, Città della Salute e della Scienza University Hospital, Turin, Italy; 8 Department of Oncology, University of Turin, Turin, Italy; 9 Centre for Medical Sciences - CISMed, University of Trento, Trento, Italy; 10 Department of Anesthesia and Intensive Care, Santa Chiara Regional Hospital, APSS Trento, Trento, Italy

**Keywords:** COVID-19, Respiratory failure, Helmet-CPAP, Non-rebreather mask, Venturi mask, Respiratory effort, Lung stress, Gas-exchange, Hemodynamics

## Abstract

**Background:**

in COVID-19 acute respiratory failure, the effects of CPAP and FiO_2_ on respiratory effort and lung stress are unclear. We hypothesize that, in the compliant lungs of early Sars-CoV-2 pneumonia, the application of positive pressure through Helmet-CPAP may not decrease respiratory effort, and rather worsen lung stress and oxygenation when compared to higher FiO_2_ delivered via oxygen masks.

**Methods:**

In this single-center (S.Luigi Gonzaga University-Hospital, Turin, Italy), randomized, crossover study, we included patients receiving Helmet-CPAP for early (< 48 h) COVID-19 pneumonia without additional cardiac or respiratory disease. Healthy subjects were included as controls. Participants were equipped with an esophageal catheter, a non-invasive cardiac output monitor, and an arterial catheter. The protocol consisted of a random sequence of non-rebreather mask (NRB), Helmet-CPAP (with variable positive pressure and FiO_2_) and Venturi mask (FiO_2_ 0.5), each delivered for 20 min. Study outcomes were changes in respiratory effort (esophageal swing), total lung stress (dynamic + static transpulmonary pressure), gas-exchange and hemodynamics.

**Results:**

We enrolled 28 COVID-19 patients and 7 healthy controls. In all patients, respiratory effort increased from NRB to Helmet-CPAP (5.0 ± 3.7 vs 8.3 ± 3.9 cmH_2_O, p < 0.01). However, Helmet’s pressure decreased by a comparable amount during inspiration (− 3.1 ± 1.0 cmH_2_O, p = 0.16), therefore dynamic stress remained stable (p = 0.97). Changes in static and total lung stress from NRB to Helmet-CPAP were overall not significant (p = 0.07 and p = 0.09, respectively), but showed high interpatient variability, ranging from − 4.5 to + 6.1 cmH_2_O, and from − 5.8 to + 5.7 cmH_2_O, respectively. All findings were confirmed in healthy subjects, except for an increase in dynamic stress (p < 0.01). PaO_2_ decreased from NRB to Helmet-CPAP with FiO_2_ 0.5 (107 ± 55 vs 86 ± 30 mmHg, p < 0.01), irrespective of positive pressure levels (p = 0.64). Conversely, with Helmet’s FiO_2_ 0.9, PaO_2_ increased (p < 0.01), but oxygen delivery remained stable (p = 0.48) as cardiac output decreased (p = 0.02). When PaO_2_ fell below 60 mmHg with VM, respiratory effort increased proportionally (p < 0.01, r = 0.81).

**Conclusions:**

In early COVID-19 pneumonia, Helmet-CPAP increases respiratory effort without altering dynamic stress, while the effects upon static and total stress are variable, requiring individual assessment. Oxygen masks with higher FiO_2_ provide better oxygenation with lower respiratory effort.

*Trial registration* Retrospectively registered (13-May-2021): clinicaltrials.gov (NCT04885517), https://clinicaltrials.gov/ct2/show/NCT04885517.

**Supplementary Information:**

The online version contains supplementary material available at 10.1186/s13613-023-01202-0.

## Background

Randomized controlled trials have suggested that CPAP may reduce the rate of intubation in COVID-19, without affecting mortality or length of stay [1–4]. However, these studies did not assess the effects of CPAP on respiratory effort and lung stress, which may contribute to the progression of lung injury [5, 6].

Theoretically, CPAP has the potential to alleviate respiratory effort and lung stress in acute respiratory failure (ARF) [7, 8], by improving oxygenation [9, 10] and lung recruitment [11]. However, early COVID-19 pneumonia may be characterized by ventilation-perfusion inequalities [12], with little alveolar collapse and, hence, low recruitability [13, 14]. In this context, CPAP may induce overdistention [6], whereas a higher FiO_2_ may provide adequate oxygenation and avoid potentially harmful effects of positive pressure.

Indeed, the few prospective investigations available in early COVID-19 pneumonia have suggested that CPAP does not reduce respiratory effort, nor the total lung stress [6, 15, 16]. However, CPAP was not compared with lower degrees of respiratory support (*e.g.*, oxygen masks), and the isolated effects of FiO_2_ titration were not evaluated.

Here, in patients with early COVID-19 pneumonia, we used Helmet-CPAP and oxygen masks to investigate the effects of positive pressure and FiO_2_ on respiratory effort, lung stress, gas exchange and hemodynamics. We hypothesized that Helmet-CPAP would not reduce respiratory effort, and rather worsen lung stress and oxygenation when compared to oxygen masks with higher FiO_2_.

## Methods

### Experimental setting

**Fig. 1:**
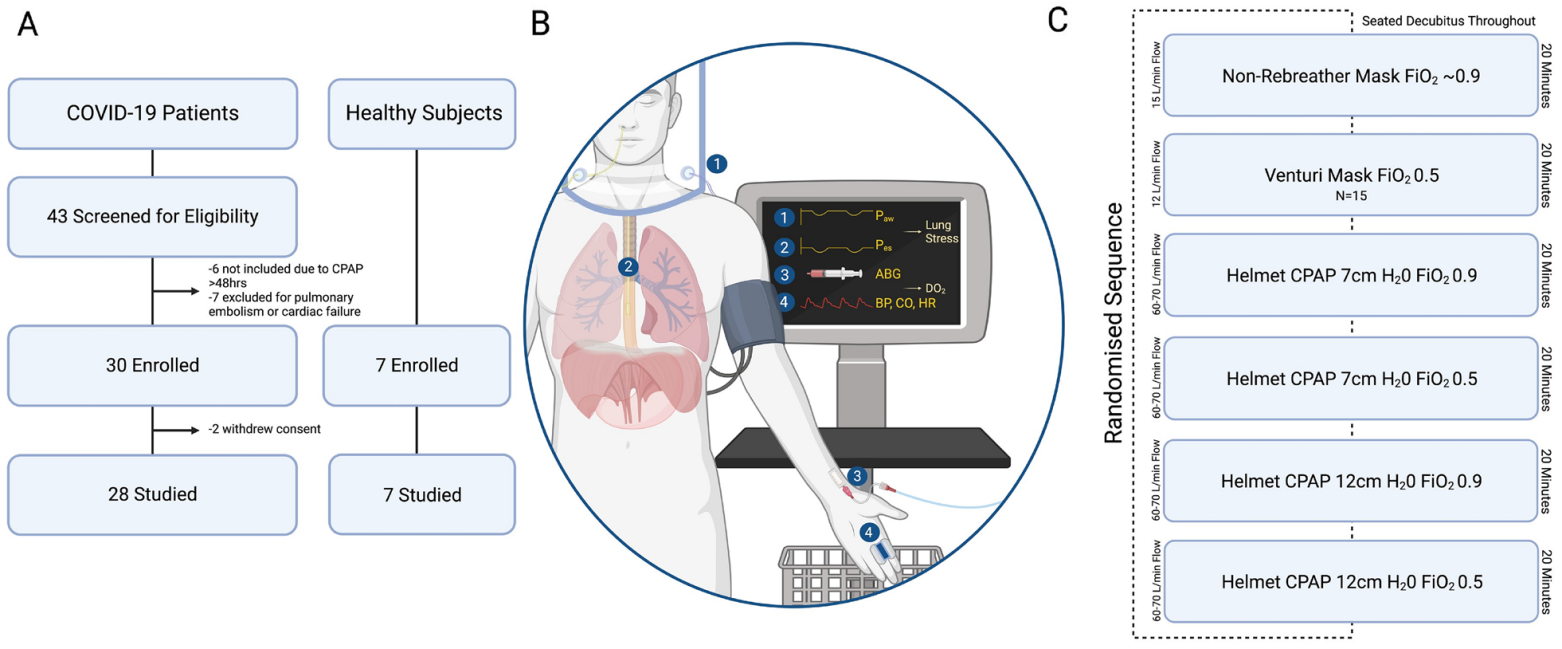
Experimental procedure. **A**: Enrolment flowchart. Of note, 12 of the 28 COVID-19 patients were recruited prior to trial registration, but with ethics approval; **B**: experimental equipment and monitoring of (1) Helmet’s pressure (P_aw_) and (2) esophageal pressure (P_es_) through OptiVent^™^ monitor, (3) arterial blood gases (ABG) through radial line, (4) blood pressure (BP), cardiac output (CO) and heart rate (HR) through CNAP^®^ monitor consisting of a finger cuff and a brachial cuff; Panel **C**: experimental protocol

This study was conducted in the COVID-19 High-Dependency Unit (HDU) of the University Hospital San Luigi Gonzaga, Orbassano-Turin (Italy) from February 1st to June 30th, 2021. Ethical approval (San Luigi Gonzaga 1565/2021) and trial registration (clinicaltrials.gov: NCT04885517) were obtained. The experimental procedure is summarized in Fig. 1.

### Inclusion and exclusion criteria

We included all confirmed SARS-CoV-2 patients with interstitial pneumonia (chest X-Ray or CT scan) who had been commenced on Helmet-CPAP by the treating clinicians within < 48 h (early pneumonia). We excluded patients with severe chronic pulmonary or cardiac disease, concomitant bacterial pneumonia, lobar or segmental pulmonary embolism at CT scan, or patients requiring immediate intubation (Additional file). Healthy volunteers were included as control group. All participants were non-sedated and provided written informed consent prior to enrolment.

### Experimental equipment and monitoring systems

Participants were equipped with:

- A nasogastric catheter with an esophageal balloon (NutriVent^™^) advanced 35–40 cm from the nostrils and filled with 4 ml of air as per manufacturer instructions.
- A non-invasive device (CNAP^®^ CNSystems-Medizintechnik-GmbH) for continuous arterial pressure and cardiac output measurement.
- A radial artery catheter for blood-gas analysis (patients only).

### Experimental protocol

In the seated decubitus (trunk at 60°, legs down at 45°), participants underwent a random sequence of 20 min steps:

1. Non-rebreather mask (NRB), 15 L/min, FiO_2_ ~ 0.9
2. Helmet-CPAP, 7 cmH_2_O, FiO_2_ 0.9, 60–70 L/min
3. Helmet-CPAP, 7 cmH_2_O, FiO_2_ 0.5, 60–70 L/min
4. Helmet-CPAP 12 cmH_2_O, FiO_2_ 0.9, 60–70 L/min
5. Helmet-CPAP 12 cmH_2_O, FiO_2_ 0.5, 60–70 L/min
6. Venturi mask (VM), 12 L/min, FiO_2_ ~ 0.5 (performed in only a subset of patients)

The levels of Helmet-CPAP (7 and 12 cmH_2_O) reflected our institution clinical guidance.

As all relevant variables except for the arterial oxygen tension (PaO_2_) and saturation (SaO_2_) were not significantly different between steps 2,3,4 and 5 (Additional file 1: Table S1), results related to these steps will be averaged and presented as a single step named “Helmet-CPAP”. PaO_2_ and SaO_2_ will be also presented relatively to single steps.

### Measurements and calculations

The naso-gastric catheter (NutriVent™) and the Helmet (Dimar s.r.l.) were connected to a monitoring system (OptiVent™) continuously displaying esophageal and airway pressures. To ensure reproducibility, the esophageal balloon was deflated and reinflated before each measurement, and cardiac artifacts on the esophageal trace were sought to confirm its correct placement. At the end of each step, a stable breathing pattern for at least 2 minutes was sought before freezing the OptiVent^™^ monitor (Additional file 1: Figure S1). Esophageal and airway pressures were measured in five consecutive breaths and subsequently averaged. The work of breathing (WOB) scale [17], the Borg’s dyspnea scale [18], hemodynamics and blood gases were concomitantly evaluated.

Respiratory effort, the inspiratory Helmet’s pressure drop, and dynamic stress were computed, respectively, as the tidal swings in esophageal, airway, and their difference, i.e., the transpulmonary pressure [19]. The static stress associated with Helmet-CPAP was calculated as the change in end-expiratory transpulmonary pressure from NRB [20, 21]. Total stress was the sum of static and dynamic stress [6]. Oxygen delivery (DO_2_) was calculated from cardiac index and the arterial oxygen content [22]. All equations are reported in the Additional file 1.

### Study outcomes

The main outcome was the effect of positive pressure (NRB vs Helmet-CPAP) on respiratory effort and lung stress. Secondary outcomes were the effects of positive pressure on gas-exchange and hemodynamics, and the isolated effects of FiO_2_ (NRB vs VM) on the same variables.

### Sample size

Due to the physiological design of the study, and to the lack of comparable investigations at the time it was performed, a formal sample size was not calculated. Consistent with similar physiological studies [15, 16, 23, 24], we aimed to recruit a convenience sample size of 30 patients and 7 healthy controls.

### Statistical analysis

Data are presented as mean ± standard deviation (SD). Normality was assessed with histograms and QQ plots, sphericity with Mauchly’s Tests. The effects of positive pressure (NRB vs Helmet-CPAP) and FiO_2_ (NRB vs VM) were assessed with paired Student’s t-test or Wilcoxon signed rank test as appropriate. Multiple steps were compared with one-way repeated measures ANOVA or its nonparametric equivalent the Friedman test. Appropriate post-hoc tests were corrected for multiple comparisons using Holm’s p adjustment method. Pearson’s r coefficient of linear regressions was used to evaluate correlations between variables. Two-sided p values < 0.05 were considered statistically significant. R studio version 4.2.2 was used for statistical analysis.

## Results

A flowchart describing patients’ enrolment is available in Fig. 1. As shown, among the 30 patients enrolled, 2 withdrew their consent because of discomfort, thereby 28 were eventually studied. Their characteristics are reported in Table 1. All 7 healthy controls were successfully studied. This group will be described separately.

**Table 1:** Characteristics of COVID-19 patients (N = 28)

Anthropometric measures	
Age	65 ± 10
Female sex, N (%)	8 (29)
BMI, kg/m^2^	28.8 ± 4.2
Comorbidities	
Smoke history, N (%)	14 (50)
Hypertension, N (%)	19 (68)
Diabetis mellitus, N (%)	6 (2)
COPD, N (%)	0 (0)
Congestive heart failure, N (%)	0 (0)
Chronic kidney disease, N (%)	0 (0)
COVID-19 history	
Days from symptoms onset	9 ± 3
Days from hospital admission	3 ± 5
Days from oxygen support	3 ± 3
Days from CPAP	1 ± 1
Previous awake proning, N (%)	11 (39)
Parameters at enrolment	
SOFA score	3 ± 1
CPAP (cmH_2_O)	10 ± 1
FiO_2_	0.5 ± 0.1
SpO_2_	95 ± 5
Respiratory rate (bpm)	24 ± 5
WOB scale^a^	3 ± 1
Laboratory data	
White blood cells (10^9^/L)	8.2 ± 3.2
Hemoglobin (g/dL)	13.9 ± 1.5
Platelet count (10^3^/mcL)	214 ± 80
D dimer (ng/mL)	919 ± 487
C-reactive protein (mg/L)	7.9 ± 5.1
Creatinine (mg/dL)	0.9 ± 0.2
Outcome	
ICU admission, N (%)	9 (32)
Endotracheal intubation, N (%)	8 (29)
In hospital death, N (%)	6 (21)

*BMI* body mass index, *COPD* chronic obstructive pulmonary disease, *SOFA* sequential organ failure assessment, *CPAP* continuous positive airway pressure, *FiO*_*2*_ fraction of inspired oxygen, *SpO*_*2*_ peripheral oxygen saturation, *ICU* intensive care unit

^a^No patients showed nasal flaring, two had evident expiratory abdominal contraction, one demonstrated palpable inspiratory contraction of the sternocleidomastoid muscle

### Effects of positive pressure (NRB vs Helmet-CPAP)

#### Respiratory effort and lung stress

**Fig. 2:**
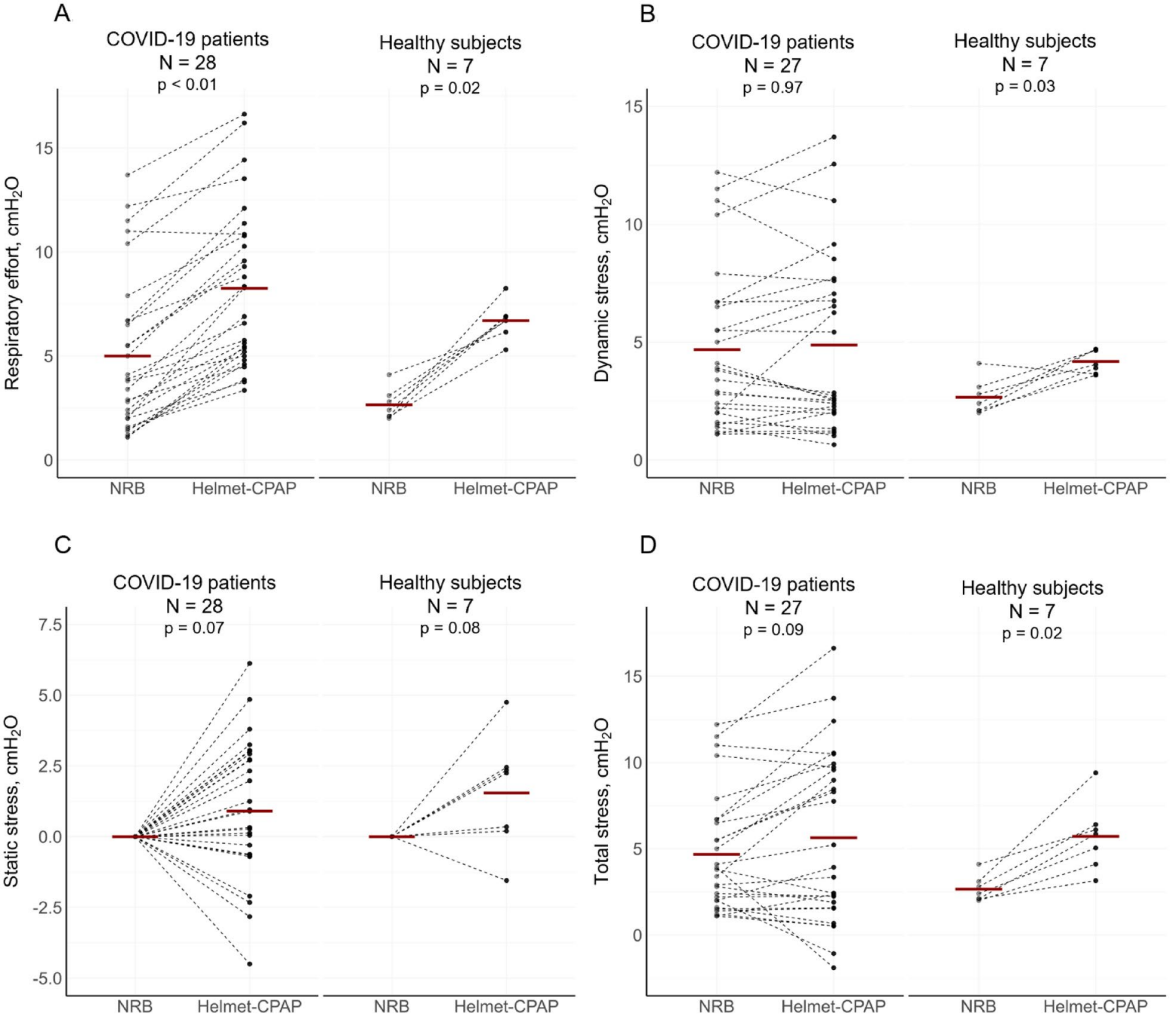
Effects of positive pressure on respiratory effort and lung stress. Differences in respiratory effort (**A**), dynamic **B**, static **C** and total lung stress **D** between NRB and Helmet-CPAP in COVID-19 patients and Healthy subjects. Of note, measurement of airway pressure was missing in one patientBlack dots: single patients; Red bars: mean values; *NRB* Non-rebreather mask

As shown in Table 2 and Fig. 2A, the esophageal swing increased in all but one patient from NRB to Helmet-CPAP (5.0 ± 3.7 vs 8.3 ± 3.9 cmH_2_O, p < 0.01), while clinical signs of effort (*i.e.*, respiratory rate, the WOB scale, and the Borg dyspnea scale) were not affected by positive pressure. The increase in esophageal swing (3.3 ± 1.5 cmH_2_O) was paralleled by a comparable (p = 0.16) inspiratory drop in Helmet’s pressure (− 3.1 ± 1.0 cmH_2_O, Additional file 1: Figure S1), thereby dynamic stress remained stable (Fig. 2B). Similarly, the static lung stress did not significantly change from NRB to Helmet-CPAP, but a high variability was observed: the change ranged from − 4.5 to + 6.1 cmH_2_O, with a decrease in 10 patients (36%) and an increase in 18 patients (64%) (Fig. 2C). The total lung stress showed similar variability (range of change from − 5.8 to + 5.7 cmH_2_O), remaining overall stable from NRB to Helmet-CPAP (Fig. 2D).

**Table 2:** Effects of positive pressure (NRB vs Helmet-CPAP)

Group	COVID-19 patients (N = 28)			Healthy controls (N = 7)		
Respiratory support	NRB	Helmet-CPAP	*p*	NRB	Helmet-CPAP	*p*
Respiratory effort, cmH_2_O	5.0 ± 3.7	8.3 ± 3.9	< 0.01	2.7 ± 0.8	6.7 ± 0.9	0.02
Helmet’s pressure drop, cmH_2_O	0 ± 0	−3.1 ± 1.0	< 0.01	0 ± 0	-2.5 ± 0.6	< 0.01
Dynamic stress, cmH_2_O	5.0 ± 3.8	4.9 ± 3.8	0.97	2.7 ± 0.8	4.2 ± 0.5	0.03
Static stress, cmH_2_O	0 ± 0	0.9 ± 2.4	0.07	0 ± 0	1.5 ± 2.0	0.08
Total stress, cmH_2_O	5.0 ± 3.8	5.7 ± 5.0	0.09	2.7 ± 0.8	5.7 ± 2.0	0.02
Respiratory Rate, bpm	25 ± 5	25 ± 5	0.33	14 ± 2	14 ± 5	0.93
Work of breathing Scale (WOB)	2 ± 1	3 ± 1	0.37	1 ± 0	2 ± 0	< 0.01
Borg dyspnea scale	0 ± 0	0 ± 0	0.16	0 ± 0	0 ± 0	> 0.99
PaO_2_, mmHg	107 ± 55	156 ± 57^a^	< 0.01	/	/	/
SaO_2_, %	95 ± 6	96 ± 4	< 0.01	/	/	/
PaCO_2_, mmHg	36 ± 4	36 ± 3	0.23	/	/	/
pH	7.46 ± 0.02	7.46 ± 0.03	0.19	/	/	/
Systolic blood pressure, mmHg	136 ± 22	134 ± 19	0.54	116 ± 15	129 ± 11	0.06
Diastolic blood pressure, mmHg	77 ± 15	79 ± 12	0.50	70 ± 8	87 ± 10	0.02
Heart Rate, bpm	75 ± 14	74 ± 12	0.85	73 ± 9	75 ± 14	0.83
Cardiac index, L/min/m^2^	3.2 ± 0.7	2.9 ± 0.5	0.02	3.4 ± 0.6	3.2 ± 0.5	0.09
Oxygen delivery, ml/min/m^2a^	577 ± 125	555 ± 115	0.18	/	/	/

^a^See Fig. 3 for details about single Helmet-CPAP steps with different positive pressures and FiO_2_. Measurement of airway pressure was missing in one patient

*NRB* non-rebreather mask, *CPAP* continuous positive airway pressure, *PaO*_*2*_ partial pressure of arterial oxygen, *SaO*_*2*_ percentage of oxygen saturated hemoglobin in arterial blood, *PaCO*_*2*_ partial pressure of arterial carbon dioxide

#### Determinants of respiratory effort and lung stress

During NRB, respiratory effort was higher in patients with lower PaO_2_ (Additional file 1: Figure S2). The increase in respiratory effort from NRB to Helmet-CPAP correlated with the inspiratory Helmet’s pressure drop (Additional file 1: Figure S3). Conversely, neither the PaO_2_ nor the respiratory effort during NRB predicted changes in respiratory effort and lung stress due to the application of Helmet-CPAP (Additional file 1: Figure S4).

#### Gas exchange and hemodynamics

**Fig. 3:**
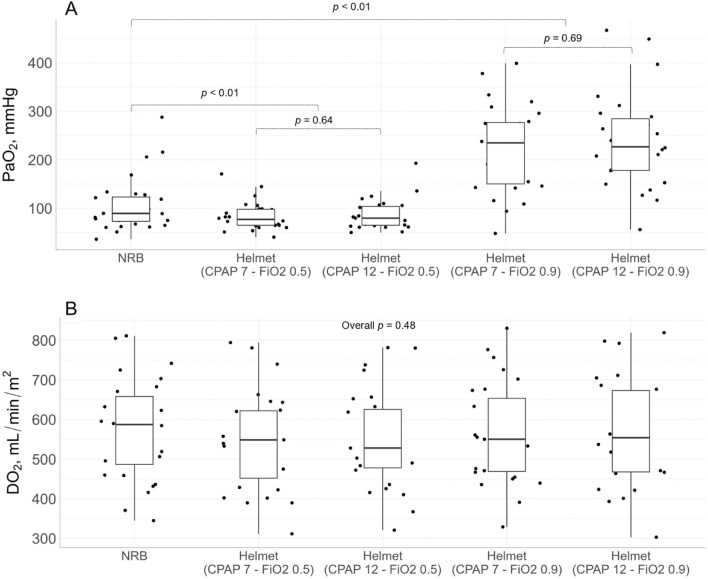
Changes in PaO_2_ and DO_2_ with positive pressure and FiO_2_. Changes in PaO_2_ **A** and DO_2_ **B** between steps in COVID-19 patients; The overall *p* value for the change in PaO_2_ between the five steps was < 0.01 (not shown). Black dots: single patients; Boxplots: medians and interquartile ranges; *PaO*_*2*_ arterial oxygen tension. *NRB* Non-rebreather mask. *DO*_*2*_ oxygen delivery

PaO_2_ decreased from NRB (107 ± 55 mmHg) to Helmet-CPAP with FiO_2_ 0.5 (86 ± 30 mmHg), while it increased with Helmet-CPAP with FiO_2_ 0.9 (232 ± 92 mmHg). Positive pressure levels (7 vs 12 cmH_2_O) did not affect the PaO_2_ (Fig. 3A). Cardiac index significantly decreased from NRB to Helmet-CPAP (Table 2), thereby oxygen delivery remained unchanged even when PaO_2_ increased (Fig. 3B).

### Effects of FiO_2_ (NRB vs VM)

**Fig. 4:**
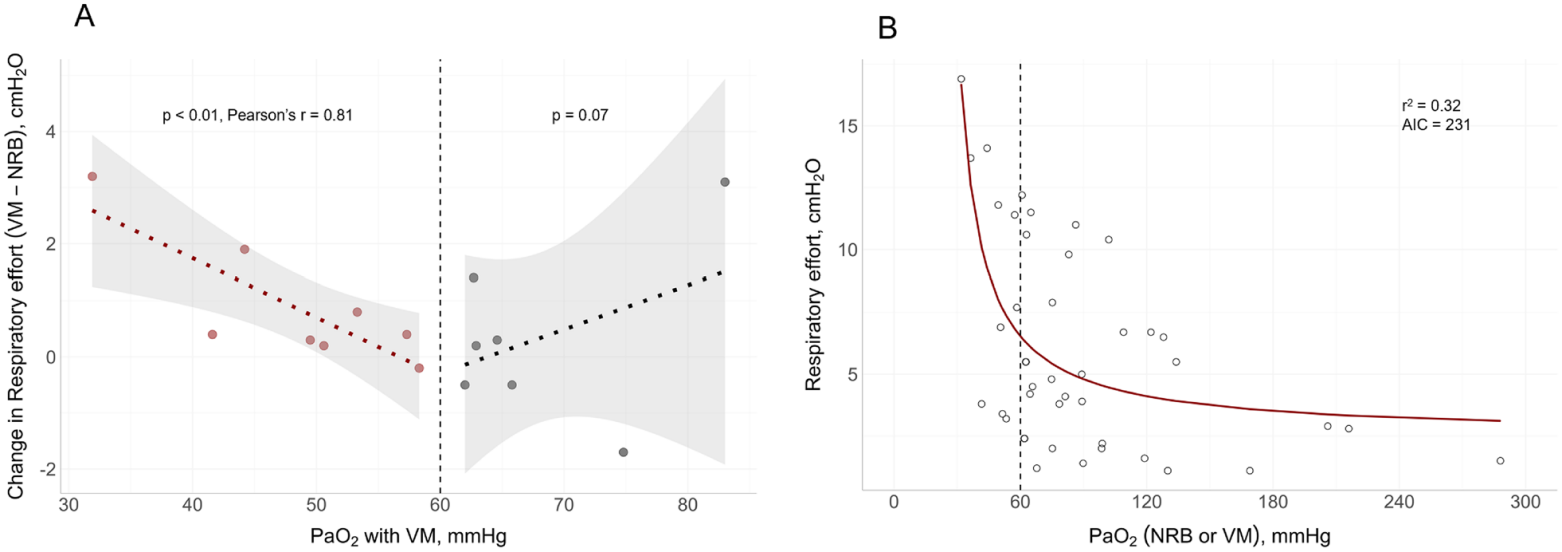
Relationship between oxygenation and respiratory effort. **A**: relationship between PaO_2_ during VM, and the change in respiratory effort from VM to NRB (n = 15): only in patients with PaO_2_ < 60 mmHg (n = 8, red dots), a strong, significant relationship was found. **B**: relationship between PaO_2_ and respiratory effort with oxygen masks (NRB and, when available, VM); PaO_2_: arterial oxygen tension. *NRB* Non-rebreather mask, *VM* Venturi mask, *AIC* Akaike information criterion

The VM step was available in 15 patients. Compared to NRB, PaO_2_ decreased, while respiratory effort, lung stress, and hemodynamics remained overall stable (Additional file 1: Table S2). However**,** when PaO_2_ fell below 60 mmHg, we observed an increase in respiratory effort proportional to the degree of hypoxemia (Fig. 4A).

### Healthy controls

The seven healthy controls had a mean age of 30 ± 2 years, 2 (29%) were female, BMI was 22.2 ± 2.9 kg/m^2^. Table 2 and Fig. 2 report their response to Helmet-CPAP: the esophageal swing increased by 4.1 ± 1.3 cmH_2_O, and an inspiratory Helmet’s pressure drop was observed in all participants. The total lung stress increased by 3.1 ± 1.7 cmH_2_O, due to an increase in dynamic stress with an overall stable, but highly variable, static stress. The Helmet elicited expiratory abdominal contraction in 5 subjects, increasing the WOB scale. Cardiac output did not change with Helmet-CPAP.

## Discussion

In this physiological study we investigated the effects of positive pressure and FiO_2_, delivered with Helmet-CPAP and oxygen masks, on respiratory effort, lung stress, gas-exchange and hemodynamics in early COVID-19 pneumonia. We found that:

1. Helmet-CPAP increases respiratory effort without altering dynamic stress;
2. The effect of Helmet-CPAP on static and total stress is highly variable;
3. High FiO_2_ has a greater impact than positive pressure on oxygenation;
4. FiO_2_ affects respiratory effort only in the most hypoxic conditions.

### Helmet-CPAP increases respiratory effort without altering dynamic stress

To the best of our knowledge, this is the first study reporting a systematic increase in respiratory effort associated with the application of Helmet-CPAP. Furthermore, by measuring Helmet’s pressure, we could demonstrate a dissociation between the increased respiratory effort and an unaltered dynamic stress. This finding is new, as the two variables are generally considered equivalent during CPAP [6, 8, 15].

While the inability of Helmet-CPAP to reduce dynamic stress was previously reported [6, 15, 16], and likely reflects low potential for lung recruitment in early COVID-19 pneumonia [13, 14], the increase in esophageal swing is less intuitive. A possible explanation is that the Helmet added an inspiratory load by not maintaining its pressure throughout the respiratory cycle [25, 26]. Indeed, to generate pressure in our Helmets, we used spring-loaded adjustable valves, which have been shown to cause airway pressure instability, similar to the one we observed in this study [27]. Interestingly, Menga et al. reported an increasing trend in respiratory effort with Helmet-CPAP compared to high flow nasal oxygen, but airway pressure was not monitored [15]. Conversely, no other study investigating the effects of Helmet-CPAP on respiratory effort had a baseline esophageal swing available for comparison, as patients kept breathing through the Helmet even when CPAP was set at *zero* cmH_2_O [6, 16].

Although it would be tempting to solely ascribe the increase in esophageal swing to the concomitant inspiratory decrease in Helmet’s pressure, the correlation between the two variables was rather weak (r = 0.36, Additional file 1: Figure S2). Another contributing factor may be expiratory muscle activation to limit increases in end expiratory lung volume (EELV) [28–31]. Indeed, relaxation of expiratory muscles at the onset of inspiration would increase the esophageal swing, mimicking inspiratory effort [32]. Although a gastric pressure trace would be required for confirmation, the lower than expected static stress associated with Helmet-CPAP in our patients might support this hypothesis (see below).

### The effect of Helmet-CPAP on static and total stress is highly variable

Static stress represents the static transpulmonary pressure associated with CPAP. It has been previously estimated from passive properties of the respiratory system (normal lung to chest wall elastance ratio: E_R_ = 0.7) [6, 33]. However, in spontaneously breathing patients with possible expiratory muscles activation [28–31, 34], direct assessment of changes in end-expiratory transpulmonary pressure (P_L(exp)_), a proxy of changes in EELV [20, 21], might be a better option. Using this method, we found that Helmet-CPAP did not change static stress as would be predicted from the passive properties of the respiratory system (Additional file 1: Figure S5). Moreover, we observed a high variability between patients: some decreased or did not change static stress, suggesting expiratory muscle activation “protecting” their EELV [25, 28, 35]; others increased static stress, suggesting an increase in EELV. Such variability reflected on the total lung stress, which, overall, remained stable from NRB to Helmet-CPAP. This is at variance with previous studies estimating P_L(exp)_ from passive properties of the respiratory system and concluding that Helmet-CPAP inevitably increased the static, and thereby total lung stress [6]. Our findings suggest that the effects of Helmet-CPAP on P_L(exp)_ should be directly measured, as a high variability exists at a single patient level. Moreover, the response to Helmet-CPAP in terms of lung stress did not seem predictable from the baseline oxygenation or respiratory effort in our patients (Additional file 1: figure S4), further stressing the need for individualized assessment.

### High FiO_2_ has a greater impact than positive pressure on oxygenation

The preponderant role of FiO_2_ over positive pressure in improving oxygenation in our patients is depicted in Fig. 3. Indeed, PaO_2_ decreased from NRB to Helmet-CPAP with FiO_2_ 0.5, and was not affected by increasing CPAP levels. This is in line with ventilation-perfusion inequalities as major contributors to hypoxemia [12, 36], and with low potential for lung recruitment [13, 14] at this disease stage. Moreover, even with the higher PaO_2_ reached during Helmet-CPAP with FiO_2_ 0.9, the concomitant decrease in cardiac output blunted any increase in oxygen delivery [22]. Taken together, these findings suggest that oxygen masks with high FiO_2_ provide better oxygenation than Helmet-CPAP in early COVID-19 pneumonia.

### FiO_2_ affects respiratory effort only in the most hypoxic conditions

By altering FiO_2_ in the absence of positive pressure (NRB vs VM), we observed an increase in respiratory effort only when PaO_2_ fell below 60 mmHg (Fig. 4A). This supports that hypoxic drive plays a role only at very low oxygen tensions [9, 10, 37]. Indeed, the roughly hyperbolic relationship between respiratory effort and PaO_2_ in our patients (Fig. 4B) resembles that obtained from carotid bodies in vitro [10] (details can be found in the Additional file). Interestingly, despite the very low PaO_2_ reached during the VM step, no patient reported dyspnea, in line with the observed “silent hypoxemia” of early COVID-19 pneumonia [38].

### Healthy subjects

The vast majority of our findings were confirmed in healthy subjects. Namely, the increase in esophageal swing, the inspiratory Helmet’s pressure drop, and the variable change in static stress with Helmet-CPAP. The visible contraction of abdominal muscles further suggests expiratory activation. The only notable difference in healthy controls was a significant increase in dynamic stress with Helmet-CPAP, suggesting that positive pressure might have increased their tidal volume, as previously reported [24, 39, 40].

### Strengths and limitations

Strengths and novelties of this study are (1) the baseline step with oxygen masks, allowing detection of the inspiratory load added by the Helmet; (2) the contemporaneous measurement of esophageal and airway pressure, differentiating respiratory effort from dynamic stress during Helmet-CPAP; (3) the direct measurement of end-expiratory transpulmonary pressure to evaluate the static stress associated with Helmet-CPAP; (4) the simultaneous evaluation of blood gases and cardiac output to assess the effects of Helmet-CPAP on oxygen delivery; (5) the inclusion of a control group of healthy subjects. Limitations include the small sample size which, however, was similar to previous studies [15, 16, 23], in keeping with the complexity of these experiments. Other limitations are the lack of assessment of static lung volumes, tidal volume, inspiratory flow and gastric pressure, and the limited duration of the protocol steps (20 min): although this is similar to previous studies [6, 16], adaptation to CPAP (for example, reducing expiratory muscles activation) may require more time.

## Conclusions

In early COVID-19 pneumonia, Helmet-CPAP increases respiratory effort, likely due to airway pressure instability during inspiration. A higher FiO_2_ with oxygen masks provides better oxygenation with lower respiratory effort. The dynamic lung stress is not reduced by Helmet-CPAP, questioning its role in alleviating lung injury at this disease stage. The response of static and, thereby, total lung stress to Helmet-CPAP is highly variable, and cannot be predicted from the passive properties of the respiratory system. Multimodal monitoring of esophageal and airway pressure, blood gases and cardiac output allows thorough evaluation of the appropriateness of respiratory support at a single patient level.

### Supplementary Information

**Additional file 1: Figure S1.** Esophageal and airway pressure measurement. **Figure S2.** Relationship between PaO_2_ and respiratory effort with NRB. **Figure S3.** Relationship between the Helmet’s inspiratory pressure drop and the increase in respiratory effort from NRB to Helmet-CPAP. **Figure S4.** Relationship between PaO_2_ or respiratory effort with NRB and changes in respiratory effort or lung stress from NRB to Helmet-CPAP. **Figure S5.** Measured *vs* estimated static stress. **Table S1.** Invariance of variables during Helmet-CPAP steps. **Table S2.** Effects of FiO_2_ (NRB vs VM).

## Data Availability

The dataset is available from the corresponding author on reasonable request.

## Abbreviations

CPAP: Continuous positive airway pressure
FiO_2_: Inspired oxygen fraction
NRB: Non-rebreather mask
VM: Venturi mask
COVID-19: Coronavirus infectious disease-19
ARF: Acute respiratory failure
EELV: End expiratory lung volume
E_R_: Elastance ratio
BMI: Body mass index
COPD: Chronic obstructive pulmonary disease
SOFA: Sequential organ failure assessment
SpO_2_: Peripheral oxygen saturation
ICU: Intensive care unit
WOB: Work of breathing
PaO_2_: Partial pressure of arterial oxygen
SaO_2_: Percentage of hemoglobin saturated with arterial oxygen
PaCO_2_: Partial pressure of arterial carbon dioxide
CI: Cardiac index
DO_2_: Oxygen delivery
