# Supplementary material for: Effects of CPAP and FiO2 on respiratory effort and lung stress in early COVID-19 pneumonia: a randomized, crossover study
Source: Ann Intensive Care. 2023 Oct 17;13:103. doi: 10.1186/s13613-023-01202-0 (PMC10581975; doi:10.1186/s13613-023-01202-0)
Supplement: Supplementary file 1 — Additional file 1: Figure S1. Esophageal and airway pressure measurement. Figure S2. Relationship between PaO2 and respiratory effort with NRB. Figure S3. Relationship between the Helmet’s inspiratory pressure drop and the increase in respiratory effort from NRB to Helmet-CPAP. Figure S4. Relationship between PaO2 or respiratory effort with NRB and changes in respiratory effort or lung stress from NRB to Helmet-CPAP. Figure S5. Measured vs estimated static stress. Table S1. Invariance of variables during Helmet-CPAP steps. Table S2. Effects of FiO2 (NRB vs VM). [file 13613_2023_1202_MOESM1_ESM.docx]

**Effects of CPAP and FiO_2_ on respiratory effort and lung stress in early COVID-19 pneumonia: a randomized, crossover study.**

Lorenzo Giosa, Patrick Duncan Collins, Martina Sciolla, Francesca Cerrone, Salvatore Di Blasi, Matteo Maria Macrì, Luca Davicco, Andrea Laguzzi, Fabiana Gorgonzola, Roberto Penso, Irene Steinberg, Massimo Muraccini, Alberto Perboni, Vincenzo Russotto, Luigi Camporota, Giacomo Bellani, Pietro Caironi.

Additional file Material

**Additional file Methods**

Criteria for impending intubation:

- pH<7.30 with PaCO2 >45
- Bradypnea (respiratory rate <12 bpm)
- Paradoxical abdominal breathing
- Mental status alteration

**Additional file Equations**

Respiratory mechanics

Respiratory effort, Helmet’s pressure drop during inspiration, and dynamic stress (all in cmH_2_O) were computed, respectively, as the tidal swings (∆) in esophageal (P_es_), airway (P_aw_) and transpulmonary pressure (P_L_):

**Equation 1**

$${\Delta P}_{\mathrm{es}}=P_{es (exp)}- P_{es (insp)}$$

**Equation 1**

$${\Delta P}_{\mathrm{aw}}=P_{aw (exp)}- P_{aw (insp)}$$

**Equation 2**

$${Dynamic stress=\Delta P}_{L}= {\Delta P}_{\mathrm{es}}- {\Delta P}_{\mathrm{aw}}$$

where P_es(exp)_ and P_aw(exp)_ are the expiratory esophageal and airway pressure immediately before the inspiratory drop, while P_es(insp)_ and P_aw(insp)_ are the inspiratory nadirs. P_aw(exp)_ and P_aw(insp)_ were considered *zero* during oxygen masks.

The static stress (cmH_2_O) due to the application of positive pressure was considered *zero* during oxygen masks, and calculated as the change in end-expiratory transpulmonary pressure (∆P_L(exp)_) during Helmet-CPAP:

**Equation 3**

$$P_{L(exp)}=P_{aw (exp)}- P_{es (exp)}$$

**Equation 4**

$$Static stress= {\Delta P}_{L(exp)}$$

Where ∆ is the difference between P_L(exp)_ at the applied CPAP and that measured during oxygen masks.

Total stress (cmH_2_O) was calculated as the sum of dynamic and static stress.

Hemodynamics and arterial gases

Oxygen delivery (DO_2_) in mL/min/m^2^ was computed as

**Equation 5**

$\mathrm{DO}_{2}=CI \cdot[1.34 \cdot Hb \cdot\mathrm{SaO}_{2}+\left( 0.003 \cdot\mathrm{PaO}_{2} \right)]$

Where CI is cardiac index (CNAP®) in dL/min/m^2^, Hb is hemoglobin in g/dL, 1.34 is its oxygen carrying capacity in mL/g, SaO_2_ is its saturation fraction, PaO_2_ is the arterial partial pressure of oxygen in mmHg, and 0.003 is its solubility constant in mL/mmHg/dL.

Additional file **Discussion**

Details on Figure 4-Panel B

The fitted hyperbola has the following equation:

$$y= \frac{a\cdot x}{x-b}$$

Where y is the respiratory effort (esophageal swing) and x is the arterial oxygen tension (PaO_2_). The coefficients *a* and *b* that best fitted the data were found to be 3.6 (95% CI 2.95-4.38; p value <0.001) and 25.3 (95% CI 21.71-27.37; p value <0.001), respectively. The fitted hyperbola had an r squared (r^2^) of 0.32, and the lowest Akaike information criterion (AIC = 231) compared to other curves we attempted to fit to the data.

Additional file **1: Figures**

*Additional file* **1: Figure S1. Esophageal and airway pressure measurement.**


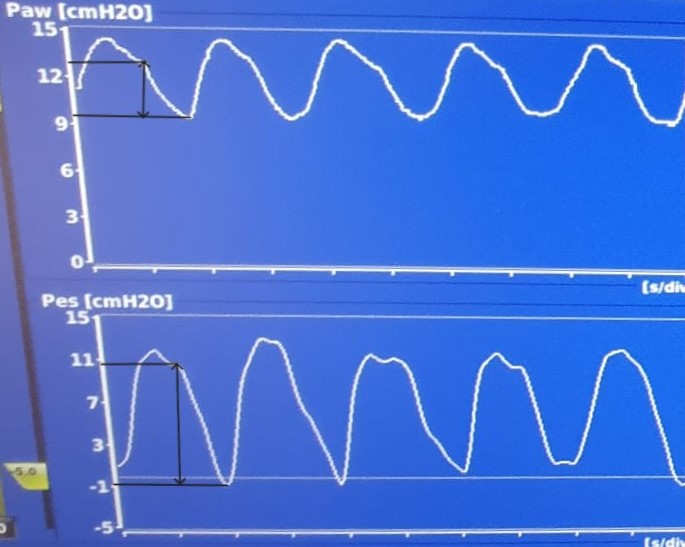


Example of a typical airway (top curve, P_aw_) and esophageal (bottom curve, P_es_) pressure tracing as displayed by the OptiVent™ monitor once frozen after 2 minutes of stable breathing pattern at the end of a protocol step. As shown, in the first recorded breath, at a set Helmet-CPAP of 12 cmH_2_O, this patient had an esophageal pressure swing (∆P_es_) of ~ 12 cmH_2_O, and a concomitant inspiratory drop in airway pressure (∆P_aw_) of ~ 3.5 cmH_2_O. Five consecutive breaths were evaluated and averaged for each patient at each step.

**Additional file1: Figure 2. Relationship between PaO_2_ and respiratory effort with NRB**


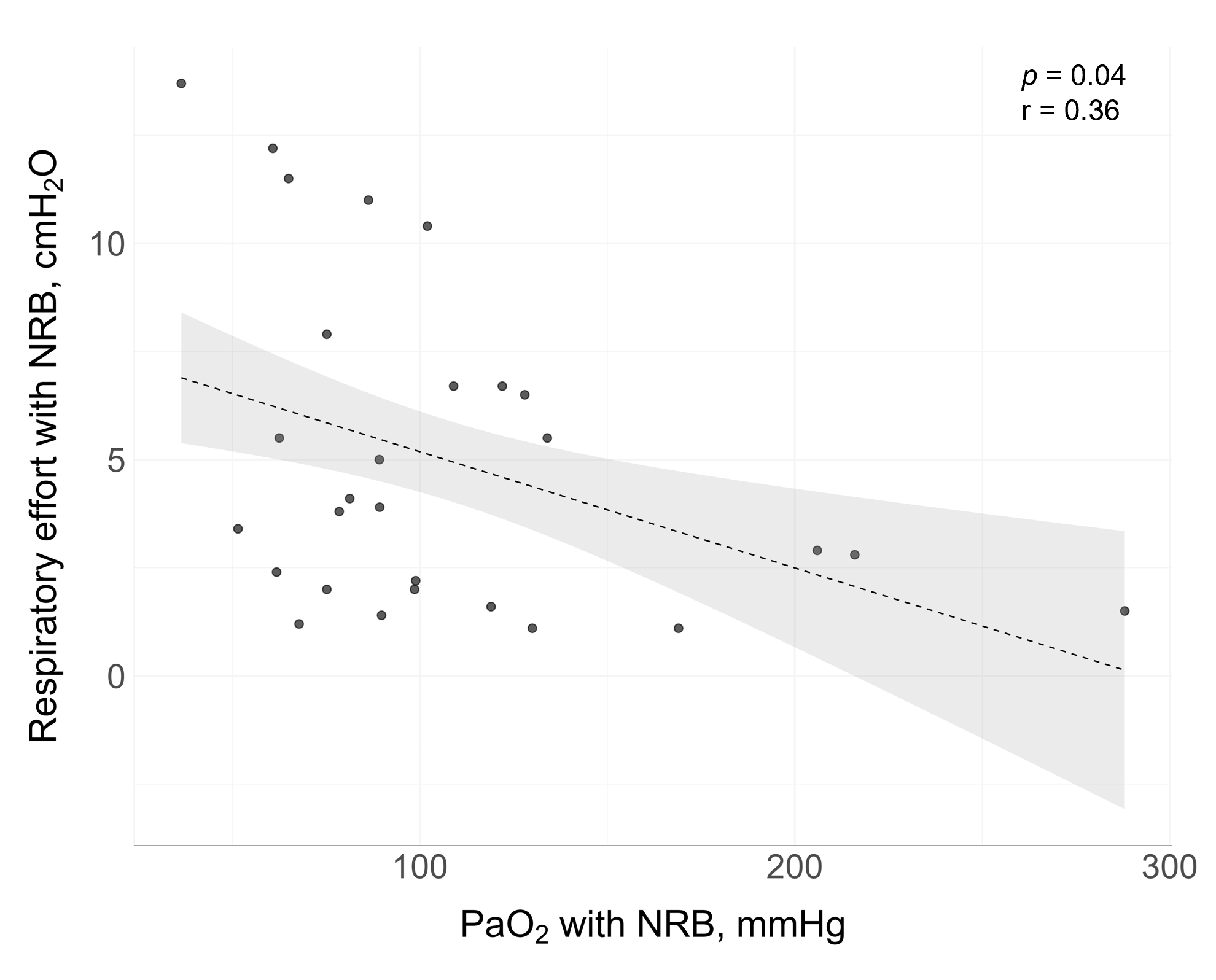
NRB: non-rebreather mask; PaO_2_: arterial oxygen tension.

**Additional file1: Figure 3. Relationship between the Helmet’s inspiratory pressure drop and the increase in respiratory effort from NRB to Helmet-CPAP.**

**
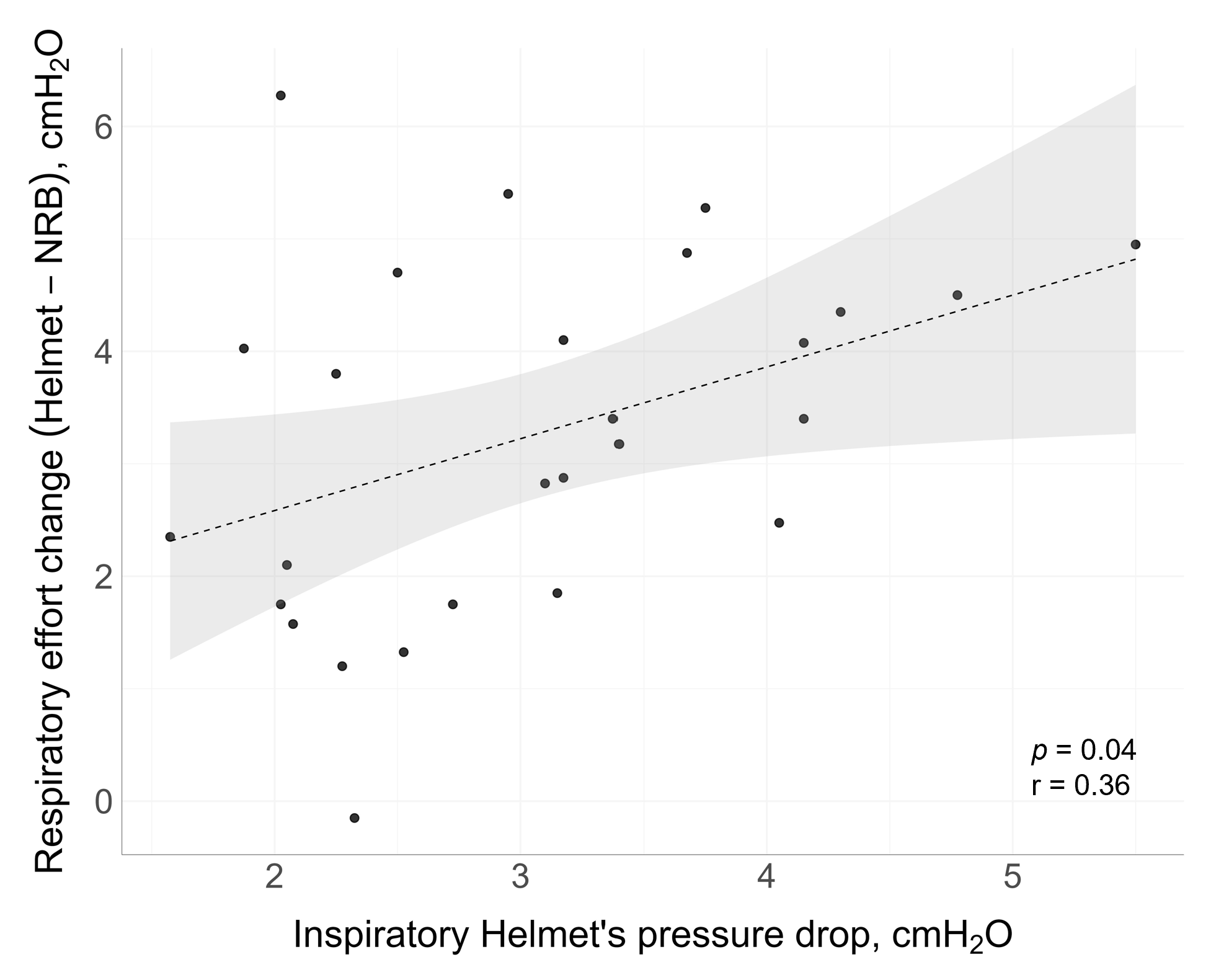
**NRB: non-rebreather mask.

**Additional file1: Figure 4. Relationship between PaO_2_ or respiratory effort with NRB and changes in respiratory effort or lung stress from NRB to Helmet-CPAP.**


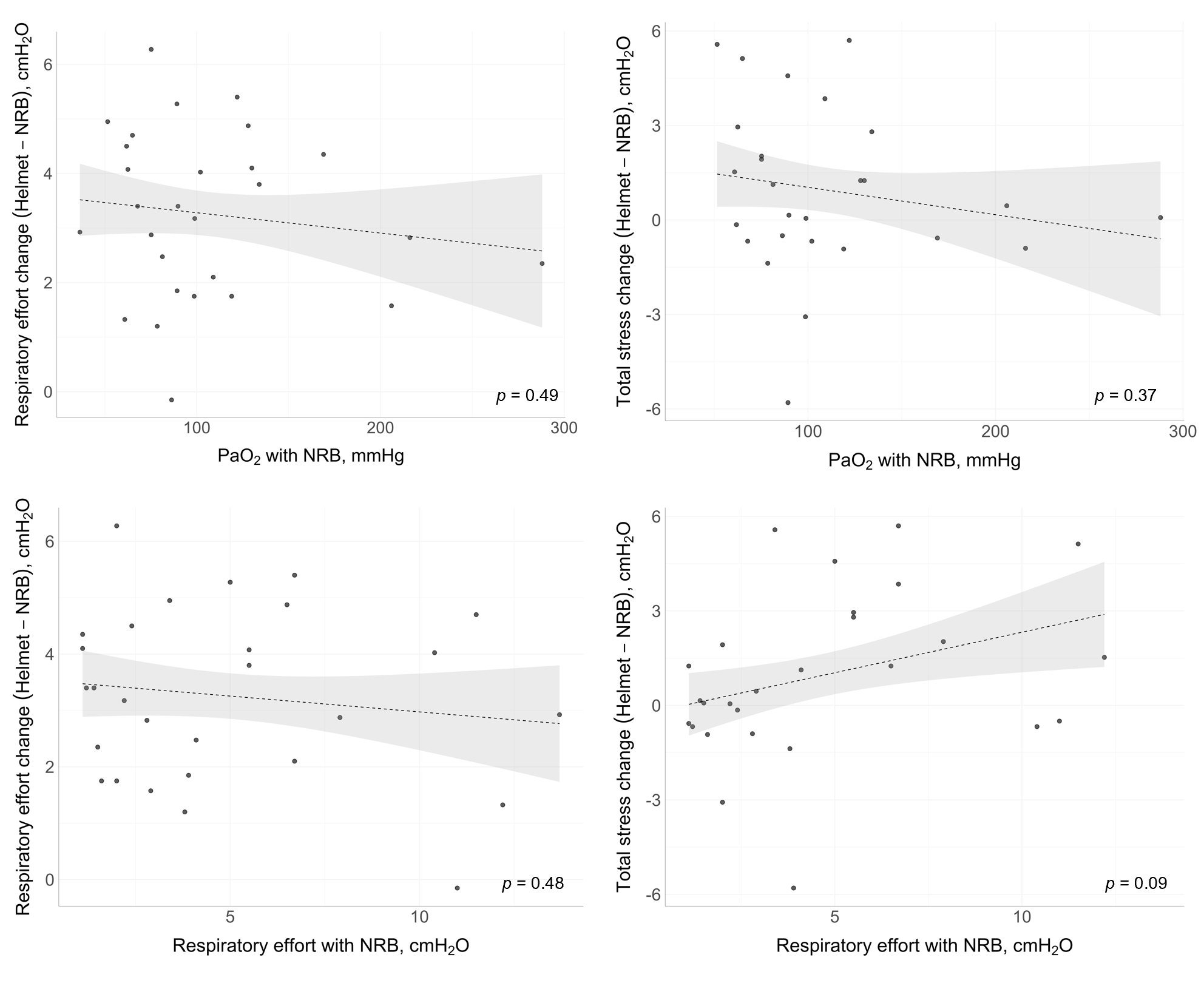
NRB: non-rebreather mask; PaO_2_: arterial oxygen tension.


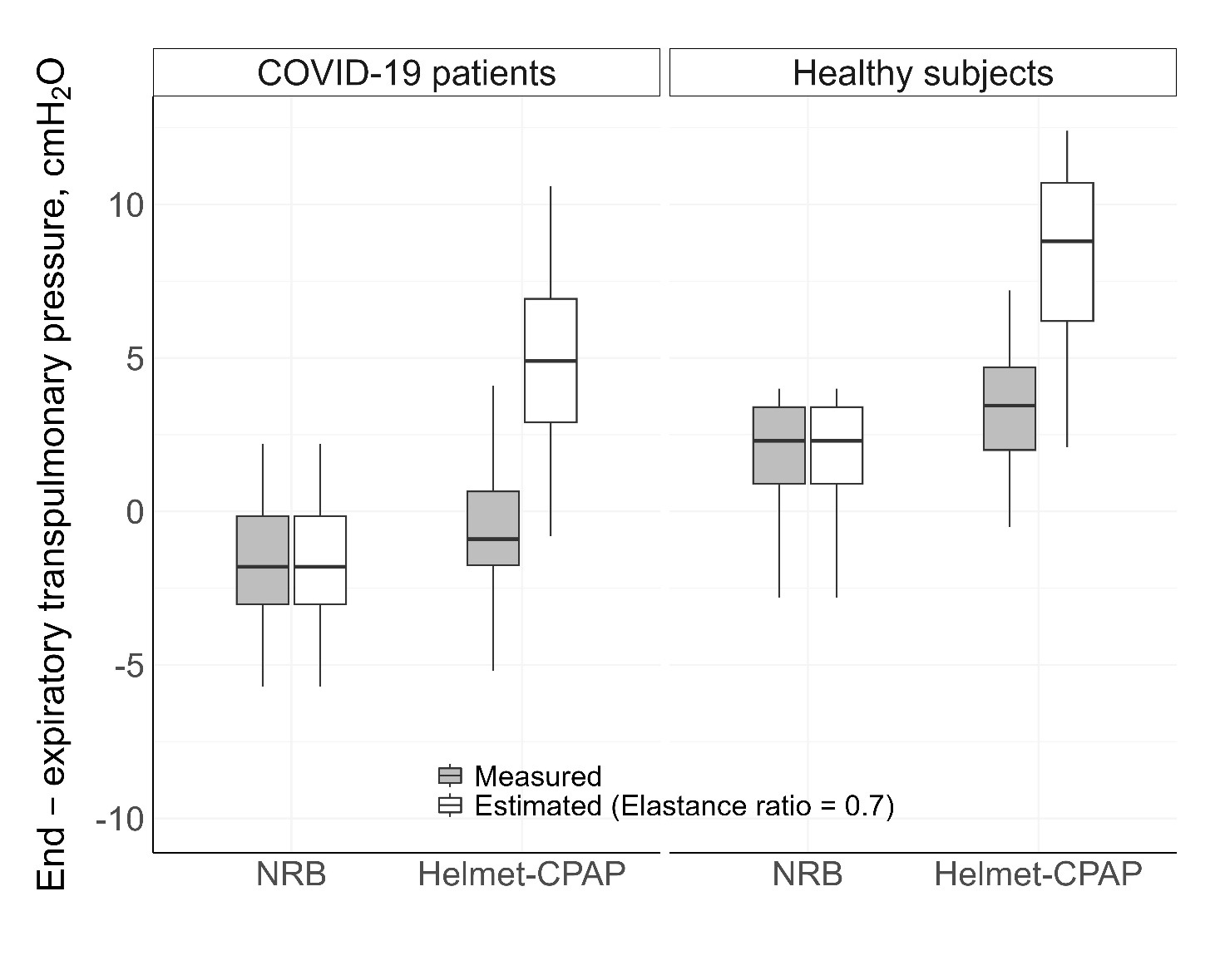
**Additional file1 Figure 5. Measured *vs* estimated static stress.**

Measured values of end-expiratory transpulmonary pressure (P_L (exp)_) are depicted in grey, estimated values in white: calculation of the estimated P_L (exp)_ was performed assuming a constant ratio of elastances equal to 0.7: P_L (exp)_ = P_L (exp)_ with NRB + CPAP∙0.7. As shown, the measured change in P_L (exp)_ (i.e., the static stress) was lower than its value estimated from passive properties of the respiratory system.

NRB: non-rebreather mask; CPAP: continuous positive airway pressure.

**Additional file1: Tables**

**Additional file1: Table 1. Invariance of variables during Helmet-CPAP steps**

| Step (CPAP-FiO_2_) | 7-0.5 | 7-0.9 | 12-0.5 | 12-1 | *p* |
| --- | --- | --- | --- | --- | --- |
| Respiratory effort, cmH_2_O | 7.7 ± 3.7 | 8.4 ± 4.4 | 8.1 ± 3.8 | 8.8 ± 4.2 | 0.06 |
| Dynamic stress, cmH_2_O | 4.9 ± 3.9 | 5.3 ± 4.4 | 4.6 ± 3.4 | 4.7 ± 3.8 | >0.99 |
| Total stress, cmH_2_O | 5.4 ± 4.9 | 5.3 ± 4.9 | 5.8± 5.2 | 6.0 ± 5.3 | 0.21 |
| Respiratory rate, bpm | 24 ± 5 | 25 ± 5 | 24 ± 6 | 25 ± 6 | 0.59 |
| PaCO_2_, mmHg | 36 ± 4 | 36 ± 4 | 36 ± 4 | 36 ± 4 | 0.22 |
| Cardiac index, ml/min/m^2^ | 3.0± 0.5 | 2.9 ± 0.6 | 3.0 ± 0.6 | 2.9 ± 0.6 | 0.26 |

CPAP: continuous positive airway pressure; FiO_2_: fraction of inspired oxygen; PaCO_2_: partial pressure of arterial carbon dioxide.

**Main message**: this table shows that variables were not affected by changes in positive pressure and/or FiO_2_ levels during Helmet-CPAP. For this reason, we decided to group these steps into a single one named “Helmet-CPAP”.

**Additional file1: Table 2. Effects of FiO_2_ (NRB vs VM)**

| Respiratory support | NRB | VM | *p* |
| --- | --- | --- | --- |
| Respiratory effort, cmH_2_O | 7.2 ± 3.7 | 7.8 ± 4.4 | 0.12 |
| Respiratory Rate, bpm | 26 ± 5 | 25 ± 5 | 0.60 |
| WOB scale | 2 ± 1 | 2 ± 1 | >0.99 |
| Borg dyspnea scale | 0 ± 1 | 0 ± 1 | >0.99 |
| PaO2, mmHg | 91 ± 41 | 58 ± 13 | <0.01 |
| SaO_2_, % | 94 ± 8 | 88 ± 9 | <0.01 |
| PaCO_2_, mmHg | 36 ± 5 | 35 ± 4 | <0.01 |
| Lactate, mMol/L | 1.1 ± 0.5 | 1.2 ± 0.4 | 0.63 |
| pH | 7.46 ± 0.02 | 7.47 ± 0.03 | <0.01 |
| Systolic blood pressure, mmHg | 135 ± 21 | 128 ± 18 | 0.04 |
| Diastolic blood pressure, mmHg | 71 ± 16 | 68 ± 19 | 0.28 |
| Heart Rate, bpm | 76 ± 14 | 78 ± 14 | 0.01 |
| Cardiac index, L/min/m^2^ | 3.4 ± 0.8 | 3.5 ± 0.9 | 0.67 |
| Oxygen delivery, ml/min/m^2^ | 585 ± 135 | 567 ± 163 | 0.38 |

This table refers to the 15 patients undergoing a step with Venturi mask.

NRB: non-rebreather mask; VM: Venturi mask; WOB: work of breathing; PaO_2_: partial pressure of arterial oxygen; SaO_2_: percentage of oxygen saturated hemoglobin in arterial blood; PaCO_2_: partial pressure of arterial carbon dioxide.

**Main message**: this table shows that, despite worsening oxygenation, reducing FiO_2_ with oxygen masks did not overall affect respiratory effort, lung stress and hemodynamics. However, an increase in respiratory effort was observed when PaO_2_ fell below 60 mmHg with VM (**Figure 4**). Of note, despite the profound hypoxemia during VM, no patient reported dyspnea, and the oxygen delivery remained high, thereby no lactate was produced (see Discussion for further details).
